# Advances in time-sensitive assessment and intervention for acute stress reaction

**DOI:** 10.3389/fpsyg.2026.1836560

**Published:** 2026-08-17

**Authors:** Huiling Rao, Li Ding, Yushu Wang, Xiaomei Ren

**Affiliations:** 1 Department of Medical Engineering, The First Affiliated Hospital (Southwest Hospital) of Army Medical University, Chongqing, China; 2 Department of Emergency Medicine, The First Affiliated Hospital (Southwest Hospital) of Army Medical University, Chongqing, China

**Keywords:** acute stress reaction, early assessment, psychological intervention, time-sensitive intervention, trauma exposure

## Abstract

Acute stress reaction (ASR) is a trauma-sensitive, time-dependent response that should be distinguished from acute stress disorder (ASD), post-traumatic stress disorder (PTSD), and PTSD-risk constructs. Given the narrow window for early psychological care, this narrative review summarizes evidence on time-sensitive assessment and intervention across three post-trauma periods: the immediate aftermath, the acute phase (24–72 h), and the subacute phase (3 days to 1 month). Because instruments specifically validated for ASR are limited, the review also draws on evidence from acute traumatic stress symptoms, ASD screening, and PTSD-risk monitoring while interpreting these constructs separately. Based on available evidence and expert synthesis, we propose a preliminary staged framework. In the immediate period, identification should prioritize safety, orientation, communication capacity, and basic functioning. During the acute phase, symptom screening should combine quantitative tools with risk stratification. During the subacute phase, re-evaluation should focus on symptom trajectories, functional recovery, and referral decisions. Immediate-response approaches, including Psychological First Aid, PIE-based strategies, the 6C model, YaHaLOM, and iCOVER, may provide practice-informed strategies for stabilization, orientation, and restoration of perceived control; however, their evidence base derives mainly from guidelines, field experience, training evaluation, and implementation studies rather than clinical outcome trials. For selected high-risk individuals with persistent symptoms or marked functional impairment, trauma-focused cognitive behavioral therapy may offer clinical benefit, with selective use of eye movement desensitization and reprocessing, medication, mindfulness-based approaches, or social support as appropriate adjuncts. Wearable monitoring, ecological momentary assessment, and digital phenotyping may support dynamic monitoring but should remain adjuncts to clinical judgment. Current evidence is limited by military-derived models, methodological heterogeneity, scarce long-term functional outcomes, and insufficient validation in civilian healthcare settings. Future research should test and refine integrated pathways aligning assessment, stratified intervention, and longitudinal monitoring with critical post-trauma windows.

## 1 Introduction

Acute stress reaction (ASR) refers to a transient physiological and psychological response that occurs when an individual perceives an immediate threat or demand. It is characterized by marked activation of the sympathoadrenal medullary system and the hypothalamic–pituitary–adrenal (HPA) axis, which mobilizes the body's energy resources to meet urgent situational demands (Cadeddu et al., 2025; Russell and Lightman, 2019). In the 11th revision of the International Classification of Diseases (ICD-11), ASR is defined as “transient emotional, somatic, cognitive, or behavioral symptoms occurring immediately after exposure to an extremely threatening or horrifying event” (Adler and Gutierrez, 2022a). By contrast, the Diagnostic and Statistical Manual of Mental Disorders does not define the concept of ASR, but instead defines acute stress disorder (ASD) as a condition in which an individual directly experiences, witnesses, or learns that a close family member or friend has been exposed to a traumatic event, such as death, serious injury, or sexual violence, and subsequently develops intrusion symptoms, negative mood, dissociative symptoms, avoidance symptoms, and arousal symptoms lasting from 3 days to 1 month and causing clinically significant distress (American Psychiatric Association, 2022). Post-traumatic stress disorder (PTSD) is diagnosed when these symptoms persist for more than 1 month, whereas delayed expression of PTSD refers to cases in which the full diagnostic criteria are not met until at least 6 months after the traumatic event.

Several studies have suggested that ASR may precede the development of ASD or PTSD; however, experiencing ASR does not necessarily lead to subsequent ASD or PTSD symptoms (Adler and Gutierrez, 2022a). Nevertheless, evidence indicates that distress during trauma is associated with an increased risk of PTSD, and that the first 2 weeks after trauma represent a critical window for interventions aimed at preventing or reducing the risk of PTSD (Kessler et al., 2021; Qi et al., 2025). Review evidence from military settings suggests that untreated or insufficiently managed combat-related acute stress reactions may be associated with elevated long-term PTSD risk, although the underlying cohorts differ in definitions, follow-up duration, and effect-size reporting (Adler and Gutierrez, 2022a).

## 2 Scope and methods

Relevant literature was identified through searches of major bibliographic databases, including PubMed, Web of Science, Google Scholar, PsycINFO, Embase, and Scopus. Additional records were identified by manually screening the reference lists of key reviews and clinical guidelines. Searches covered publications from database inception to 31 December 2025. The search focused primarily on English-language publications. Two non-English-language articles were included because they addressed topics directly relevant to acute stress responses or early intervention and could not be adequately replaced by English-language sources: one Chinese-language article on combat stress responses (Zhengzhi and Jia, 2019) and one Hebrew-language article on therapeutic interventions for acute stress disorder (Kalla et al., 2024). Search strategies combined controlled vocabulary terms and free-text keywords related to acute stress reaction, acute stress response, acute stress disorder, post-traumatic stress, early assessment, screening, psychological first aid, crisis intervention, trauma-focused cognitive behavioral therapy, and digital phenotyping.

Given the heterogeneity of ASR and the breadth of the evidence base, both military and civilian studies were considered. Eligible publications addressed early post-traumatic stress responses, stage-specific assessment or intervention approaches, or emerging measurement strategies relevant to the immediate, acute, or subacute post-trauma phases. Included evidence types comprised clinical guidelines, systematic reviews, meta-analyses, and randomized or observational studies. Approximately 189 records were considered during title, abstract, or full-text screening, and 88 publications were included as core sources in the narrative synthesis. This article was designed as a narrative review rather than a systematic review; therefore, no PRISMA-based study selection process, protocol registration, or formal risk-of-bias assessment was undertaken.

Military and civilian evidence were weighted according to the clinical question addressed. Military and operational studies were used primarily to inform immediate response models, peer-based interventions, and field management principles. Civilian emergency, trauma, and healthcare studies were weighted more heavily when discussing epidemiological estimates, standardized assessment instruments, ASD and PTSD-risk monitoring, structured psychological treatment, pharmacological interventions, and referral decision-making.

## 3 Incidence of ASR and the need for timely intervention

### 3.1 Incidence of ASR in the context of war

In wartime settings, the incidence of ASR is relatively high, although substantial variation has been reported across different conflicts. Its occurrence is closely associated with multiple factors, including service branch, combat intensity, operational difficulty, duration of warfare, disparities in military strength between opposing forces, and the stage of the conflict. Historical military reports and secondary analyses have shown that in battles of low to moderate intensity, the proportion of personnel loss attributable to psychological injury ranges from 0% to 10%, whereas in conflicts of moderate or higher intensity, this proportion increases to 39%−86% (Belenky et al., 1983; Jones and Wessely, 2001). These statistics indicate that combat intensity has a profound impact on the occurrence of combat-related psychological trauma (Blood and Gauker, 1993). These figures should be interpreted as historical estimates of combat-related psychological or psychiatric casualties rather than standardized epidemiological estimates of ASR, because definitions, ascertainment methods, and combat contexts varied substantially across reports.

During World War II, stress casualties were estimated at approximately 15%−30% of those wounded in action, influenced in part by the scale and duration of the conflict (Helmus and Glenn, 2005). After World War II, the U.S. military strengthened psychological prevention and control measures for combat stress among soldiers (Yeo, 2023). In later conflicts, including the Korean War, the Vietnam War, and the Iraq War, the reported incidence of ASR appeared to be lower than that observed during World War II (Cooke, 2014). These findings suggest that pre-deployment education and psychological preparedness may help reduce the incidence of combat stress reactions and improve combat effectiveness.

### 3.2 ASD-risk estimates in non-war settings relevant to ASR management

In civilian emergency and medical settings, reported estimates relevant to early ASR management varies substantially according to trauma type, clinical context, and assessment method. Most available civilian estimates concern ASD, early post-traumatic stress symptoms, or PTSD risk rather than ICD-11 ASR itself; so, these data should be interpreted as evidence relevant to early ASR management. A meta-analysis of road traffic accident survivors included 2,989 participants, of whom 287 were identified as having ASD, and reported a pooled ASD prevalence of 15.81% (95% CI: 8.27%−25.14%), with substantial heterogeneity across studies (*I*^2^ = 96.8%, *p* < 0.001) (Dai et al., 2018). Subgroup analyses indicated significant differences by country, assessment instrument, age, and sex (Dai et al., 2018). Among young and middle-aged patients with acute myocardial infarction, a cross-sectional study of 190 patients aged 18–60 years found that 65 patients met DSM-5 criteria for ASD, corresponding to a prevalence of 34.21%. In that study, ASD scores were negatively correlated with social support, and multivariable regression identified attachment-related anxiety, infarct-related artery, perceived support, in-hospital complications, and attachment-related avoidance as major associated factors (Wu et al., 2022). A multicenter cross-sectional study of 422 adult trauma patients in Ethiopia reported an ASD prevalence of 44.15% (95% CI: 39.4%−49.0%). In multivariable logistic regression, complications (AOR = 2.22, 95% CI: 1.36–3.60), prolonged hospitalization (AOR = 1.89, 95% CI: 1.21–2.95), low social support (AOR = 3.21, 95% CI: 1.66–6.19), and moderate social support (AOR = 1.99, 95% CI: 1.14–3.48) were significantly associated with ASD (Hagos et al., 2024).

### 3.3 High-risk populations and the need for early intervention

Studies conducted in both wartime and peacetime settings have shown that not all individuals exposed to trauma develop ASR; only a subset subsequently develops ASD. From the perspective of exposure characteristics, threats that are intense, prolonged, or unpredictable are more likely to precipitate short-term emotional, cognitive, and behavioral disturbances. In combat settings, factors such as combat intensity, phase of combat, duration of exposure, and mission difficulty are associated with the occurrence of ASR. In non-war settings, life-threatening events such as medical emergencies, massive hemorrhage, and violent assault may likewise induce marked stress responses.

Cross-sectional studies of traumatized patients have identified several demographics, clinical, and psychosocial correlates of ASD, including prior psychiatric illness, trauma history, perceived stress, clinical complications, prolonged hospitalization, and inadequate social support (Hagos et al., 2024; Worku et al., 2022). For example, in the multicenter Ethiopian trauma study, complications, prolonged hospital stay, and low or moderate social support remained independently associated with ASD after multivariable adjustment (Hagos et al., 2024). These findings support the need to consider both injury-related severity and psychosocial resources when identifying high-risk individuals.

Both research findings and clinical experience indicate that the cornerstone of managing ASR is the early identification of high-risk individuals and the timely initiation of intervention. ASR is highly time-sensitive: symptoms may emerge immediately after trauma and evolve rapidly over the ensuing hours to weeks. If not recognized and managed within a critical time window, an acute stress reaction that is initially transient and potentially reversible may progress to persistent functional impairment or even longer-term trauma-related disorders. Evidence from prospective civilian trauma studies supports the clinical importance of the early post-trauma period. In the AURORA multisite longitudinal cohort, 666 emergency department patients after motor vehicle collision were followed for 8 weeks; 42.0% met the study-defined 8-week PTSD outcome based on self-report scales rather than clinician-administered diagnostic interviews. The associations of demographic or crash-related predictors with this outcome were largely mediated by peritraumatic symptoms and, to a lesser extent, 2-week ASD, suggesting that the first 2 weeks after trauma may represent an important window for risk identification and early intervention (Kessler et al., 2021). In a prospective study of 256 hospitalized trauma patients, the prevalence rates of ASD, 1-month PTSD, and 3-month PTSD were 20.7%, 19.5%, and 17.6%, respectively; ASD predicted later PTSD, and prediction improved when ASD was combined with clinical indicators such as severe injury and critical illness, with a maximum AUC of 0.827 (Qi et al., 2025). Evidence from military follow-up studies also suggests that combat stress reactions may be associated with later PTSD risk, although precise effect sizes are not consistently available across historical military cohorts (Adler and Gutierrez, 2022a).

Accordingly, the management of ASR should not be limited to passive observation of symptoms, but should instead adopt a proactive, time-sensitive approach characterized by early identification, early assessment, and early intervention (Matson et al., 2022). For high-risk individuals, preliminary identification and symptom stratification should be completed as soon as possible after the stressful event to ensure immediate safety, meet basic survival needs, and restore orientation to the current situation. On this basis, symptom presentation and the degree of functional impairment should be considered when determining whether on-site emergency support is sufficient or whether referral to a more systematic and specialized intervention team is warranted (O'Connor et al., 2024). In this sense, defining high-risk individuals is not merely a statistical issue, but also a clinically important step that directly influences subsequent management and the timing of intervention (Zatzick et al., 2021). Therefore, how to effectively identify and assess stress responses at different time points, and how to implement appropriate interventions accordingly, remain central issues in research on ASR. This narrative review provides an overview of evidence on time-sensitive assessment and intervention for ASR across the immediate, acute, and subacute phases. It specifically examines how bodily sensations, functional status, and behavioral manifestations can inform early identification, risk stratification, and the tailoring of intervention strategies for ASR. The term “embodied” is not a broad philosophical concept here, but is limited to a clinical meaning. It refers to the integration of subjective bodily sensations, observable behavioral organization, physiological arousal, and real-world functional status when assessing and supporting individuals after trauma. Accordingly, the term is used to denote somatic, behavioral, and functional indicators of acute stress responses.

## 4 Time-sensitive assessment of ASR

ASR refers to a transient, non-pathological reaction occurring immediately after trauma and is usually identified through contextual observation, brief interaction, and functional appraisal rather than through formal diagnostic scales. Most standardized instruments recommended for the acute or subacute post-trauma phases have been developed or validated against acute traumatic stress symptoms, probable ASD, or PTSD-risk endpoints rather than against ASR itself. For example, the SASRQ and ASRS mainly describe acute stress symptom profiles and multidimensional response patterns; the ASDS and ASDI are more closely aligned with ASD screening or diagnosis; and the IES-R and PCL-5 quantify post-traumatic stress symptom severity or support PTSD-risk monitoring. So, these tools are useful for symptom profiling, risk stratification, follow-up planning, and referral decision-making, but their results should not be interpreted as direct diagnostic measures of ASR.

### 4.1 On-site identification and initial screening after the event

Responses to acute stressful events are strongly time-dependent. In the immediate aftermath of trauma, the predominant manifestations are disturbances in emotion, cognition, and behavior (Adler and Gutierrez, 2022a). Evaluators should assess not only emotional distress but also physical sensations, behavioral responses, and functional status, as these indicators may signal acute dysregulation. At this stage, the primary goal is to rapidly identify warning signs, assess functional status, and determine whether urgent intervention is required (Ruzek et al., 2007). Guidelines and reviews indicate that acute stress responses fluctuate during the early post-traumatic period; therefore, psychological first aid should be preceded by prompt assessment, followed by appropriate intervention and ongoing reassessment of dynamic changes.

The most important task during initial on-site identification is to determine whether the affected individual exhibits clear risk signs requiring immediate intervention (Adler and Gutierrez, 2022a; Matson et al., 2022). Such risk signs mainly include severe confusion, disorientation to time and place, marked emotional dysregulation, psychomotor rigidity or excessive agitation, a trance-like state, and signs of panic, resistance, or even aggression. For some individuals, the immediate concern is not whether they meet the diagnostic threshold for a specific disorder, but whether acute stress has impaired their most basic capacity to understand and judge their surroundings, protect themselves, or communicate and cooperate with others (Adler and Gutierrez, 2022a). Therefore, the first step should be a rapid on-site assessment based on four key questions: Is the individual safe? Are they conscious and alert? Are they able to communicate? Are they able to follow simple instructions? These early observable signs reflect an individual's orientation, readiness to act, communication capacity, and overall functioning, and may be viewed as observable behavioral and functional manifestations of acute stress dysregulation.

Unlike structured interviews or symptom rating scales administered days or weeks after trauma, immediate post-incident assessment focuses primarily on observation and situational appraisal (Matson et al., 2022). Assessors should conduct a rapid evaluation based on direct observation and brief interaction with the affected individual to determine whether there is any disturbance of consciousness, impaired reality testing, marked emotional dysregulation, or unmet basic physiological needs. For example, the individual should be assessed for the ability to state their name and current location, describe the event, and understand and follow simple instructions (Kim and Han, 2021). Although such information does not support a formal diagnosis, it directly informs subsequent intervention by helping to identify individuals who require immediate accompaniment, reassurance, and support from those nearby, while also enabling an initial determination of whether high-risk individuals need more detailed medical evaluation or psychiatric intervention.

From the perspective of field practice, immediate post-incident assessment and early support are often implemented simultaneously rather than as separate processes (Wang et al., 2024). The strong field applicability of psychological first aid (PFA), as recommended by the World Health Organization, lies in the fact that its core principles of “look, listen, and link” inherently incorporate the key elements of initial screening. However, PFA is not a psychometric screening tool and therefore had no sample size, effect size, or measures of diagnostic accuracy. Through “look,” responders assess scene safety, basic physiological needs, and immediate risks; through “listen,” they evaluate the individual's current emotional state, communication ability, and the presence of obvious distress; and through “link,” they make a preliminary determination as to whether the individual should be connected to medical, family, or social support resources (Kim and Han, 2021). Immediate post-incident assessment should prioritize early, physically oriented observation rather than standardized symptom scales, which are often impractical at this stage. Observable somatic and behavioral cues, together with basic functional status, can help identify immediate acute stress responses, although they should not be interpreted as establishing a formal diagnosis of ASR, ASD, or PTSD.

### 4.2 Symptom screening and risk stratification in the acute phase

From 24 h to 3 days after trauma, the initial state of confusion or loss of control has generally subsided, and core acute traumatic stress symptoms may begin to emerge (Matson et al., 2022). At this stage, assessment should shift from immediate post-event safety checks and capacity evaluation to the screening of core manifestations, including intrusive memories, avoidance, dissociation, hyperarousal, and negative mood, with further symptom stratification based on the degree of functional impairment, while also attending to somatic arousal and behavioral dysregulation (Schnurr et al., 2024; Lang et al., 2024). Unlike on-site screening, assessment during the acute phase places greater emphasis on the early and relatively standardized identification of symptom onset, somatic arousal, behavioral dysregulation, and functional impairment, as well as on detecting high-risk individuals who may require continued monitoring, supportive treatment, or further referral (Jensen et al., 2022).

The Stanford ASR Questionnaire (SASRQ) is a representative instrument for comprehensive screening of acute stress responses during the acute phase (Jensen et al., 2022). This measure covers a broad range of symptoms closely related to ASR, including intrusive recollections, avoidance, dissociative symptoms, arousal symptoms, and negative emotional symptoms. Because the scale can also reflect important clinical information such as functional impairment and symptom duration, it is suitable for preliminary quantitative assessment of early post-traumatic symptoms (Cardeña et al., 2000; Lötvall et al., 2022). Psychometric validation studies and two decades of research on the SASRQ indicate that the instrument generally demonstrates acceptable reliability across diverse trauma contexts, as well as evidence of construct, convergent, discriminant, and predictive validity (Cardeña et al., 2000; Lötvall et al., 2022). The original SASRQ is a 30-item self-report scale designed to assess acute stress symptoms, including dissociation, anxiety-related symptoms, and functional impairment. However, because no universally accepted diagnostic cut-off has been established, its primary value lies in characterizing symptom profiles and assessing early symptom severity rather than serving as a stand-alone diagnostic instrument. The SASRQ captures intrusive and dissociative symptoms and also assesses arousal and functional impairment. Accordingly, it can help identify acute stress manifestations spanning subjective distress, physiological hyperarousal, and early disruption of daily functioning.

In addition to the SASRQ, other scales with different conceptual frameworks may also be used for acute-phase screening, depending on the assessment objective. The Acute Stress Response Scale (ASRS) focuses on capturing the multidimensional response profile of individuals exposed to acute stress and evaluates stress-related features across the emotional, cognitive, behavioral, and physiological domains. This scale can help identify the specific dimensions in which an individual shows prominent difficulty and may facilitate comparisons in stress response patterns across populations with different trauma severity or clinical characteristics (Gong et al., 2023). By contrast, the Acute Stress Disorder Scale (ASDS) has a stronger diagnostic-screening orientation. The original ASDS validation study showed good sensitivity and specificity for identifying ASD against the ASDI among 99 civilian trauma survivors, with sensitivity of 95% and specificity of 83%; its test–retest reliability was also strong in a separate sample of 107 bushfire survivors, with *r* = 0.94 (Bryant et al., 2000). A commonly cited ASDS total score of 56 predicted later PTSD with sensitivity of 91% and specificity of 93%, but approximately one-third of individuals scoring above this cut-off did not subsequently develop PTSD (Bryant et al., 2000). Therefore, ASDS is useful for early identification of probable ASD and PTSD risk, but its results should be interpreted together with clinical context, functional impairment, and follow-up assessment. Because its scoring indicators are relatively straightforward to interpret clinically, the ASDS is particularly useful for the early identification of suspected cases and high-risk individuals in vulnerable populations (Sanden et al., 2025).

The Impact of Event Scale-Revised (IES-R) also has substantial practical value during acute and subacute post-trauma symptom assessment (Krupelnytska et al., 2025). This instrument can be used to assess the level of subjective distress associated with a specific traumatic event during the previous week and to evaluate the severity of core trauma-related symptom clusters across three domains: intrusion, avoidance, and hyperarousal. In acute and subacute post-trauma research, the IES-R has been widely used to quantify event-related subjective distress rather than to diagnose ASD or PTSD. Its total-scale internal consistency is commonly reported to be high, and the IES-R has been widely used to screen for clinically significant post-traumatic stress symptoms; however, cut-off values vary across populations, trauma types, languages, and settings (Krupelnytska et al., 2025; Weiss, 2007). It can also serve as a longitudinal outcome measure during follow-up to track symptom trajectories over time and, when used in combination with ASD-related assessment tools, may help establish an assessment pathway for the early identification of high-risk individuals and the prediction of intermediate-term outcomes. The IES-R is not designed as a diagnostic instrument for ASD or PTSD. It is best suited to quantify symptom severity, assess subjective distress, and support symptom-based screening and stratification. However, it provides limited coverage of sleep disturbance, autonomic arousal, and early functional impairment.

At this stage, the primary focus is the differential identification and risk assessment of acute symptoms, with emphasis on early detection, early assessment, and early intervention; however, a definitive diagnosis is not necessarily required at this point (Shih et al., 2023; Brier et al., 2023). For patients assessed 1–3 days after trauma, clinicians and nurses may use instruments such as the SASRQ, ASRS, ASDS, or IES-R to obtain a preliminary evaluation of acute stress symptoms, subjective distress, and the extent of impairment in social functioning. Based on the impact of the crisis event and the individual's prior stress response, including symptom burden, physiological arousal, dissociation or behavioral disruption, and functional impairment, affected individuals can be provisionally classified into low-risk individuals who do not require intervention, individuals who require follow-up, individuals who need social support, and high-risk individuals who require psychiatric consultation (Brier et al., 2023). During this early stage, stratifying patients according to symptom burden and the degree of functional impairment can help ensure that the frequency of follow-up and the choice of treatment plan are better aligned with actual clinical needs (Schnurr et al., 2024).

### 4.3 Dynamic re-evaluation and outcome monitoring in the subacute phase

From 3 days to 1 month after a traumatic event, ASR-related symptoms may enter a transitional stage in which early symptoms may evolve into more persistent manifestations. Unlike the acute phase, which focuses primarily on symptom screening and preliminary stratification within the first 24 h to 3 days, the core task of subacute-phase assessment is no longer limited to identifying current symptoms. It involves further evaluating whether symptoms persist, whether they worsen over time, whether functional recovery is impaired, and whether changes in daily routines, behavioral regulation, and real-life functioning suggest progression from transient ASR-related symptoms toward ASD or a longer-term risk trajectory for PTSD (Brier et al., 2023).

The Acute Stress Disorder Interview (ASDI) is a clinician-administered structured interview developed for standardized ASD assessment during the early post-trauma period (Bryant et al., 1998; Garcia-Esteve et al., 2021). In the original validation study, the ASDI was assessed among 65 trauma survivors 1–3 weeks after trauma and showed good internal consistency (Cronbach's α = 0.90), sensitivity of 91%, and specificity of 93% relative to independent clinical diagnosis; test–retest reliability of severity scores was also strong (*r* = 0.88) in 60 trauma survivors reassessed within 2–7 days (Bryant et al., 1998). As a structured assessment conducted by trained professionals, the ASDI is well-suited to relatively stable time points after trauma for the standardized evaluation of symptom manifestations associated with ASD (Bryant et al., 1998). Compared with the quantitative instruments used in the previous stage, which rely primarily on self-report for initial screening, the ASDI has the advantage of reducing the bias associated with self-report alone and improving inter-rater reliability, because it allows persistent symptoms to be interpreted alongside behavioral organization and functional recovery (Kramer et al., 2023). For individuals who have undergone prior screening and are considered to have persistent symptoms, poor functional recovery, or clear warning signs, the use of a formal structured interview to confirm symptom persistence and its clinical significance can improve the accuracy of risk stratification and support more reliable decisions regarding subsequent referral or targeted intervention.

Monitoring during this stage using the PTSD Checklist for DSM-5 (PCL-5) is important; however, its scores must be interpreted in conjunction with the time elapsed since the traumatic event. The diagnostic criteria for PTSD require symptoms to persist for more than 1 month, so the PCL-5 cannot be used to determine whether an individual currently meets the diagnostic criteria for PTSD within the first month after exposure to a traumatic event (Brier et al., 2023; Bovin and Marx, 2023). Nevertheless, during the subacute phase, the PCL-5 remains a useful tool for identifying warning signs, monitoring post-traumatic stress symptom severity and PTSD-risk indicators, and providing a basis for subsequent follow-up assessment (Forkus et al., 2023). It can be used to evaluate symptom domains such as intrusive memories, avoidance, hyperarousal, emotional numbing, and functional impairment over a period of days to weeks. In situations where PTSD cannot yet be formally diagnosed during the acute stress period, early symptom monitoring with the PCL-5 can help bridge the observational gap between ASR and later PTSD diagnosis, thereby providing a more continuous basis for longitudinal assessment.

During the subacute phase, dynamic re-evaluation should address not only changes in symptom severity but also their embodied and functional impact on real-life functioning. Key aspects include whether sleep, eating, activity levels, and daily routines are gradually improving; whether avoidance, panic attacks, recurrent recollections, or emotional outbursts continue to interfere with normal life and work; whether persistent hypervigilance, attentional impairment, or dissociative symptoms hinder the individual's return to normal decision-making and social interaction; and whether available family, peer, or organizational support is sufficient to sustain the recovery process (Lang et al., 2024; Sippel et al., 2024). When persistent symptoms are accompanied by marked functional impairment, disturbance in daily routines, or ongoing behavioral dysregulation, their clinical significance is usually greater than that suggested by elevated symptom scores alone, and this may indicate the need for a higher level of intervention or treatment.

From a clinical perspective, the primary purpose of subacute-phase evaluation is to monitor symptom persistence, and to judge whether functional and behavioral recovery is occurring sufficiently to support continued observation, adjusted support, or referral to more specialized care. For those whose symptoms persist but have not progressed to the level of a disorder, the frequency of re-evaluation should be increased, and the intervention plan should be adjusted according to reassessment findings to prevent progression to chronic disorder. For patients with clear symptom progression, significant functional impairment, or a high likelihood of meeting diagnostic criteria for ASD, referral to a more specialized medical institution for further assessment and intervention is recommended (Shih et al., 2023; Brier et al., 2020).

### 4.4 Complementary value of emerging assessment techniques

Autonomic markers and wearable monitoring devices can serve as important adjuncts to ASR assessment. Autonomic and neurophysiological indicators may support risk prediction, while wearables enable continuous, non-invasive capture of parameters such as heart rate, heart rate variability, electrodermal activity (skin conductance), respiratory rate, skin temperature, and activity levels. These data help characterize physiological arousal and behavioral fluctuation after trauma and support longitudinal, person-level monitoring (Pinge et al., 2024). Compared with traditional assessment methods that rely primarily on subjective report, wearable monitoring can capture real-time fluctuations in physiological indicators under stressful conditions without substantially increasing participant burden, thereby providing more objective data for detecting short-term changes and tracking symptom dynamics. A 2024 systematic review of wearable-based stress detection reported that wearable devices have been used to collect physiological signals for stress detection and monitoring, with common analytic steps including data collection, preprocessing, feature computation, and machine-learning model training (Pinge et al., 2024). In the context of ASR, the importance of these techniques lies not in replacing clinical assessment, but in providing more timely physiological information to support continuous monitoring and post-event risk warning.

Biomarkers may serve as indicators for early risk prediction. A systematic review and meta-analysis of early physiological markers of PTSD risk, including 26 studies and 5,186 individuals, found that higher heart rate measured soon after trauma was associated with higher subsequent PTSD symptoms (*r* = 0.13), whereas cortisol (*r* = −0.07), diastolic blood pressure (*r* = −0.01), and systolic blood pressure (*r* = 0.02) were not consistently associated with later PTSD symptoms (Morris et al., 2016). These markers can be considered as surrogate indicators of autonomic and neuroendocrine dysregulation, providing alternative ideas for quantifying the somatic stress response in the early assessment after trauma. An immediate increase in resting heart rate at the time of trauma has been regarded as one of the more consistent, albeit modest, factors associated with later PTSD.

By contrast, the relationship between cortisol levels and subsequent outcomes remains less consistent, with findings varying across studies. Cortisol is a downstream marker of HPA-axis activation and reflects the neuroendocrine cascade through which hypothalamic corticotropin-releasing hormone and pituitary adrenocorticotropic hormone regulate adrenal glucocorticoid secretion. Related markers, such as DHEAS, and the cortisol/DHEAS ratio, may provide additional information on acute stress reactivity, stress-buffering capacity, and retrospective cumulative exposure (Mouthaan et al., 2014; Dutheil et al., 2021). The limited predictive performance of single-time-point salivary or serum cortisol may reflect circadian variation, differences in peri-traumatic sampling timing, sex effects, medication confounding, and the distinction between reactive and tonic HPA activity (Morris et al., 2016). Dynamic indices, including diurnal cortisol slope, and cortisol awakening response, may therefore be more informative than isolated cortisol values (Speer et al., 2019). Although a single acute cortisol measurement is insufficient for clinical risk prediction, prospective and review evidence suggests that HPA-axis dysregulation is associated with early post-traumatic symptom trajectories and established PTSD (Mouthaan et al., 2014; Speer et al., 2019).

These observations suggest that biomarkers may contribute to a hierarchical approach to PTSD risk assessment and may help objectively identify high-risk individuals for secondary prevention. However, individual biomarkers can be influenced by factors such as sampling time, measurement method, inter-individual variability, and specific contextual conditions. Therefore, their interpretation should always be integrated with the overall clinical picture and should not be considered in isolation from symptom severity and functional status.

Dynamic tracking of acute stress responses through ecological momentary assessment (EMA) and digital phenotyping represent an emerging technical approach (Hruska et al., 2025). Traditional retrospective questionnaires are prone to recall bias, whereas high-frequency self-report collected in daily life through smartphone applications can reduce this bias and capture short-term fluctuations in acute symptoms more sensitively. When these self-report data are combined with minimally intrusive passive sensing data, such as activity levels, sleep patterns, and communication behaviors, it becomes possible to continuously observe changes in individual behavior over time. This approach may facilitate earlier detection of warning signs, including worsening stress symptoms, stalled recovery, or delayed deterioration, thereby providing a more sensitive basis for clinical judgment (Williams et al., 2021). Such technologies are particularly suited to continuous follow-up from the acute to the subacute phase. By integrating individual self-reports with wearable-derived indicators of sleep, activity, and communication patterns, they link subjective distress to real-world physiological and behavioral changes, thereby providing important value for individualized assessment of physiological, behavioral, and functional changes after trauma.

Wearable devices, biomarkers, EMA, and digital phenotyping share a common advantage: they can compensate for the static, discrete, and subjective limitations of traditional rating scales. By capturing physiological activation, behavioral fluctuation, sleep and activity patterns, and short-term symptom dynamics, these techniques make assessment more closely aligned with the dynamic and time-sensitive nature of acute stress responses in real-world settings. Within a time-sensitive assessment framework, these techniques may be integrated with on-site identification, symptom screening, and subacute re-evaluation to establish a multimodal assessment pathway incorporating subjective symptoms, somatic and behavioral indicators, and longitudinal monitoring. Together, these components complement one another and provide a more comprehensive basis for risk stratification and intervention decision-making.

### 4.5 Comparative summary of commonly used assessment tools

ASR assessment tools differ in their measurement purposes, formats, and areas of emphasis. Therefore, Table 1 summarizes the main characteristics of the assessment tools discussed in this section. Assessors should select instruments cautiously according to the individual's clinical context, symptom profile, functional status, and assessment purpose.

**Table 1:** Comparative summary of commonly used assessment tools for ASR.

Instrument	Main use	Key psychometric properties	Commonly reported algorithm	Screening/diagnostic value and limitations	Original reference	Recent update reference
PFA	Immediate phase; on-site structured identification, stabilization, information gathering, and linkage to support	N/A as a psychometric scale; structured field framework rather than a standardized symptom measure	No numerical cut-off; operational logic commonly follows look–listen–link or comparable stepwise field procedures	Not a diagnostic or screening tool; useful for immediate support and functional stabilization. Recent review evidence suggests potential benefit for anxiety reduction and adaptive functioning, while evidence for PTSD and depression reduction is less consistent	Ruzek et al., 2007	Wang et al., 2024
SASRQ	Acute phase; broad self-report assessment of acute stress symptoms and functional impact	Internal consistency generally α = 0.80–0.95; test–retest reliability *r* = 0.69	No universally accepted diagnostic cut-off has been established for SASRQ. It is better interpreted dimensionally for symptom profiling and early severity appraisal rather than as a stand-alone diagnostic measure.	Better suited to symptom profiling and early severity appraisal than stand-alone diagnosis	Cardeña et al., 2000	Lötvall et al., 2022
ASDS	Acute phase; self-report screening of ASD and early PTSD risk	Internal consistency α = 0.96; test–retest reliability *r* = 0.94	Classic ASDS total score of 56 is often cited for later PTSD risk, but scores should be interpreted together with clinical context and functional impairment	Against ASDI for concurrent ASD identification, the original ASDS algorithm showed sensitivity 95% and specificity 83%. Separately, an ASDS total-score cut-off of 56 predicted later PTSD with sensitivity 91% and specificity 93%. These values refer to different outcomes.	Bryant et al., 2000	Brier et al., 2023
ASRS	Acute phase; multidimensional assessment of acute stress response profile	Original development study reported satisfactory construct validity, concurrent validity, test–retest reliability, and internal consistency, but no single set of universally cited summary indices has been adopted across settings	No widely accepted diagnostic cut-off; scores are generally interpreted dimensionally for symptom profiling and severity appraisal.	More useful for multidimensional profiling than for formal diagnostic case identification	Yang et al., 2011	Gong et al., 2023
IES-R	Acute to subacute phase; quantification of trauma-related subjective distress	Total-scale internal consistency is commonly reported to be high	Several screening thresholds have been used to identify clinically significant post-traumatic stress symptoms, but cut-off values vary across populations, trauma types, languages, and settings	Not designed as a diagnostic instrument for ASD or PTSD; best suited to quantify symptom severity, assess subjective distress, and support symptom-based screening and stratification	Weiss and Marmar, 1997	Krupelnytska et al., 2025
PCL-5	Subacute phase and follow-up; PTSD symptom monitoring and probable case finding	Internal consistency α = 0.94; test–retest reliability *r* = 0.82	Commonly recommended cut-offs vary across populations and settings; scores should not be used to diagnose PTSD within the first month after trauma	Diagnostic accuracy varies by population and criterion standard; useful for monitoring PTSD symptom severity and probable case finding, but not a substitute for clinician-administered diagnosis	Blevins et al., 2015	Forkus et al., 2023
ASDI	Subacute phase; clinician-administered structured interview for ASD diagnosis	Internal consistency α = 0.90; test–retest severity reliability *r* = 0.88	No summed screening cut-off; DSM-based structured diagnostic interview algorithm	Original validation reported Cronbach's α = 0.90 and test–retest severity reliability *r* = 0.88. Against independent DSM-IV clinical diagnosis, ASDI showed sensitivity 91% and specificity 93% for concurrent ASD diagnosis.	Bryant et al., 1998	Brier et al., 2023

This table is intended as a descriptive summary to support clinical orientation and evidence mapping. It should not be interpreted as indicating the comparative effectiveness, cost-effectiveness, or superiority of any particular assessment tool or intervention model.

## 5 Immediate interventions for ASR

### 5.1 Immediate response: PFA, PIE, 6C, YaHaLOM, and iCOVER

Immediate intervention for ASR refers to the initial supportive actions provided within the first few hours to days after a traumatic event to ensure safety, orientation, emotional stabilization, and restoration of basic functioning. The aim of intervention at this stage is not to immediately process distressing memories or further explore the traumatic experience, but rather to reduce hyperarousal as quickly as possible, minimize the risk of loss of control, and help the individual re-establish a connection with reality. In contrast to approaches centered on emotional catharsis or repeated review of traumatic details, the currently preferred strategy is to use an immediate intervention framework characterized by supportiveness, practicality, and low intrusiveness to help affected individuals regain a minimal sense of bodily control, situational orientation, and capacity for purposeful action (Ruzek et al., 2007). The evidence base for immediate response models differs substantially from that for structured psychotherapies. PFA, PIE, 6C, YaHaLOM, and iCOVER are primarily supported by guideline recommendations, field practice, training evaluations, implementation studies, and military or emergency-response experience.

Psychological first aid (PFA) is the most widely used approach in emergency response. It is designed to enable non-mental health professionals to provide immediate, humane, and practical supportive assistance during or immediately after a crisis. Its core principles are based on respect for each individual's dignity, autonomy, and cultural background, while avoiding the premature pathologization of normal responses to acute stress. The process can be summarized in three steps: look, listen, and link. “Look” refers to rapidly assessing scene safety, identifying basic physiological needs, and recognizing stress-related risks. “Listen” refers to using calm, empathic, and non-judgmental communication to reduce emotional and physiological arousal. “Link” refers to helping affected individuals obtain information, contact family members or friends, access necessary medical services, and mobilize social support resources (Ruzek et al., 2007). PFA is an immediate practice for recognizing distress through bodily presentation, behavioral disorganization, and disruption of basic functional needs. Because it can be deployed rapidly, requires relatively limited training, and is broadly applicable, PFA is particularly suitable as an immediate supportive intervention in contexts such as natural disasters, traffic accidents, public emergencies, and clinical emergencies.

Unlike the broad applicability of psychological first aid (PFA), the PIE principle places greater emphasis on helping individuals rapidly restore functioning in high-pressure tasks or highly organized settings (Zhengzhi and Jia, 2019). PIE stands for Proximity, Immediacy, and Expectancy. Its core premise is that intervention should be delivered as early as possible, in a setting as close as feasible to the site of the event or the individual's usual workplace. Through simplified and supportive measures, it aims to meet basic needs while consistently conveying positive expectations that the reaction is understandable, the symptoms are usually temporary, and functioning can be restored. In doing so, it helps reduce fixation in the sick role and promotes a rapid return to everyday functioning (Adler and Gutierrez, 2022a; Matson et al., 2022). The relevance of PIE in non-military settings lies in its function-oriented logic. In civilian medical and emergency settings, the value of PIE does not lie in directly applying the military concept of return to duty, but in translating it into support for the recovery of basic capacities such as sleep, eating, and mobility, as well as the reconstruction of daily routines and support systems. Conceptually, PIE also provides a foundation for later, more process-oriented immediate peer intervention programs.

The 6C model emphasizes immediate intervention, cognitive activation, and restoration of a sense of control (Farchi et al., 2018). It is an immediate intervention technique designed for states of acute emotional tension. Rather than encouraging emotional ventilation, its core principle is to use clear and structured communication to help affected individuals shift from helplessness, passivity, and subjective confusion toward an active and constructive problem-solving state. Its main advantage lies in rapidly interrupting uncontrolled, immobilized, and confused reactions while stimulating basic cognitive processes and preparatory action (Farchi et al., 2025). Compared with general supportive assistance, the 6C model is particularly suited to individuals who show marked paralysis, disorientation, or confusion but are still able to regain orientation through brief and simple communication.

YaHaLOM is a paradigmatic approach to immediate peer intervention. Developed by the Israel Defense Forces, it is designed to rapidly identify and address ASR among peers in high-pressure combat settings (Svetlitzky et al., 2020a). Its implementation involves several key steps, including establishing contact, providing support, asking simple questions, clarifying the sequence of events, and giving clear instructions. Through brief and structured interaction, the intervention aims to draw individuals out of a chaotic, isolated, and passive state and reorient them to the immediate situation. Its objectives are to reactivate cognitive functioning, restore temporal orientation, and rebuild an initial sense of control by guiding the individual to carry out simple instructions (Svetlitzky et al., 2020b). Owing to its structured format and rapid implementation, this approach is particularly suitable for settings with limited on-site resources and an urgent need for intervention, and can be delivered by colleagues or frontline personnel. Studies have shown that professionals trained in the YaHaLOM program demonstrate improved skills in responding to ASR, greater confidence in peer intervention, and progress in reducing mental health–related stigma (Svetlitzky et al., 2020a). These outcomes mainly reflect training-related competence and acceptability rather than clinical endpoints such as subsequent ASD incidence, symptom remission, or long-term functional recovery.

ICOVER is a further development of the YaHaLOM approach, with a more comprehensive intervention process (Adler et al., 2024). By adding a preparatory identification stage to the original framework, it forms a structured sequence of steps: Identification, Connection, Commitment, Verification, Organization, and Request for Action (Adler et al., 2020). Its core value lies in the rapid identification and engagement of individuals who are in a state of emotional turmoil or functional dysregulation, while providing support to reduce their sense of isolation. Subsequently, factual questioning is used to restore reality-based cognition and rational thinking. Clarifying the sequence of events helps reconstruct situational understanding and, in turn, guides the individual to take immediate and specific small actions, thereby regaining a sense of meaning and control over the environment (Adler and Gutierrez, 2022b). This makes iCOVER particularly compatible with a function-oriented understanding of acute stress, because the central concept of iCOVER is to bring individuals who are shut down, dissociated, or behaviorally immobilized back to the “here and now,” thereby enabling effective immediate intervention. In practice, iCOVER has shown strong generalizability and considerable practical value in the context of immediate intervention.

PFA, PIE, 6C, YaHaLOM, and iCOVER are not mutually contradictory, but rather represent different perspectives and operational approaches to the immediate intervention of ASR. Table 2 summarizes the key features, evidence levels, and primary advantages of five commonly discussed immediate response models for ASR. PFA provides a broadly applicable framework for crisis settings, whereas PIE emphasizes proximity, immediacy, and positive expectancy to promote functional recovery. The 6C model focuses on cognitive activation and restoration of control. YaHaLOM and iCOVER, by contrast, represent theoretical and practical models of peer-based intervention for high-pressure situations. Both approaches highlight their value in emergency settings such as emergency departments and disaster scenes, where the priorities are to rapidly restore a sense of safety and reality, ensure basic functional needs, and mobilize available human resources to support dynamic assessment, integrated management, and referral to other professionals when necessary.

**Table 2:** Comparison of immediate intervention models for ASR.

Intervention model	Target setting	Key components	Level of evidence	Primary advantages
PFA	Broad crisis and emergency settings, including disasters, traffic accidents, public emergencies, emergency departments, and other civilian or clinical contexts	Safety assessment; identification of basic physiological and practical needs; calm and supportive communication; connection with family, medical services, and social resources; commonly summarized as look, listen, and link	Guideline-supported and practice-oriented evidence. Widely recommended in crisis response; evidence mainly comes from guidelines, field implementation studies, and integrative reviews rather than strong RCT-based outcome evidence	Highly feasible, low-intensity, culturally adaptable, and suitable for non-specialist responders; helps avoid premature pathologization and provides immediate stabilization, basic needs support, and referral linkage
PIE	Originally military and combat settings; potentially adaptable to organized high-pressure civilian settings, emergency services, and healthcare environments	Proximity, Immediacy, and Expectancy; early intervention close to the event or usual work environment; simplified support; positive expectation of recovery; maintenance of role and function	Historical military doctrine and expert consensus with limited controlled empirical evidence. Evidence is mainly indirect and practice-based, with partial support from military stress-control literature and scoping reviews	Emphasizes rapid functional restoration, minimizes fixation in the sick role, and supports return to basic daily or occupational functioning; useful for preserving connection with routine and social roles
6C model	Acute emotional tension and high-pressure emergency situations, especially when individuals show confusion, behavioral freezing, helplessness, or impaired action readiness	Communication; Commitment; Cognition; Continuity; Challenge; Control; structured communication aimed at shifting the individual from helplessness and passivity toward cognitive activation and constructive action	Conceptual model with emerging empirical support. Evidence includes theoretical development and preliminary simulation- or training-based studies; broader clinical validation remains limited	Rapidly interrupts immobilization, confusion, and uncontrolled emotional reactions; emphasizes cognitive activation, restoration of control, and immediate adaptive action rather than emotional ventilation
YaHaLOM	High-risk military, combat, and occupational settings; peer-based response in environments with limited immediate access to mental health professionals	Establishing contact; providing support; asking simple factual questions; clarifying the sequence of events; giving clear instructions; peer-delivered rapid reorientation	Preliminary empirical and training-evaluation evidence. Evidence mainly comes from military training studies and peer-intervention evaluations; clinical outcome evidence remains limited	Suitable for peer delivery, resource-limited settings, and rapid field use; helps individuals move from chaotic, isolated, or passive states toward orientation, connection, and simple action
iCOVER	Military units, high-pressure operational environments, emergency response contexts, and potentially emergency departments or disaster scenes	Identification, Connection, Commitment, Verification, Organization, and Request for Action; identification of ASR signs; supportive connection; factual questioning; organization of event sequence; request for immediate small action	Pilot and implementation evidence. Evidence includes pilot testing, acceptability/training studies, and military adaptation reports; stronger controlled effectiveness evidence is still needed	More operationalized and stepwise than YaHaLOM; useful for individuals who are shut down, dissociated, emotionally overwhelmed, or behaviorally immobilized; supports return to the here and now, action orientation, and basic functional control

This table is a descriptive summary only; no comparative effectiveness analysis, cost analysis, or formal ranking of intervention models was performed.

### 5.2 Early support: functional recovery and referral decision-making

Within 24 to 72 h after a traumatic event, the management of acute stress should shift from immediate post-event stabilization to early support aimed at restoring functioning (Matson et al., 2022). At this stage, many affected individuals may have moved beyond the initial state of profound disorientation, rigidity, or loss of control; however, symptoms such as intrusive thoughts, avoidance behaviors, hyperarousal, sleep disturbance, emotional instability, and attentional impairment may persist. These symptoms can substantially disrupt basic daily rhythms, communication, cooperation, and practical functioning. Therefore, interventions at this stage should include physiological and behavioral stabilization and the restoration of sleep, nutrition, physical activity, daily routines, communication, and basic functional engagement, while providing appropriate psychological support to reduce the risk of role fixation and functional stagnation. This stage also provides an important opportunity to determine whether referral to specialized medical care is warranted (Cancio and Cancio, 2023).

The IMPRESS principle is an important framework for guiding early support during this stage. Derived from the PIE principle, it expands the original emphasis on immediacy, proximity, and expectancy by incorporating simplified support as a key additional component. From a function-oriented perspective, the value of IMPRESS lies in translating early support into concrete recovery tasks, such as restoring bodily regulation, daily rhythm, physical engagement, and social participation. It advocates that affected individuals rest and recover in a non-medical environment, remain within a group context, and receive multilevel supportive care. Specific measures include ensuring basic needs, such as rest, nutrition, and hydration, in a safe and comfortable setting, while enabling affected individuals to accomplish simple and feasible goals with the support of peers, supervisors, and caregivers, thereby restoring daily rhythm, sense of control, physical functioning, and social engagement (Cancio and Cancio, 2023; Cooper et al., 2024). In settings such as emergency medical rescue, disaster response, and high-risk operations, the IMPRESS model suggests that if the individual is not in immediate life-threatening danger, support should focus on restoring basic functioning rather than providing intensive and comprehensive psychological treatment. IMPRESS is better understood as a practice-oriented framework derived from military and operational stress management principles rather than as an intervention with a mature RCT-based evidence base. Therefore, quantitative estimates of its independent effects on ASD outcomes are not consistently available.

The BICEPS principle is another intervention model commonly used during the early support phase of acute stress management. It emphasizes six core elements: brevity, immediacy, avoidance of fixation in the sick role, expectancy, proximity, and simplicity (Cooper et al., 2024). According to this principle, intervention should be delivered as early as possible, within the shortest feasible time frame, and through simple methods that can be implemented by frontline personnel. Support should be provided in a non-hospital setting with minimal medicalization, thereby reducing stigma and preventing individuals from becoming entrenched in the patient role (Cooper et al., 2024). If the individual remains at least partially cooperative and shows no clear risk of self-harm, harm to others, or severe psychiatric symptoms, initial support may be provided in a more familiar environment or through the person's existing support system, rather than through immediate transfer to a highly medicalized setting. This approach can reduce additional psychological burden, preserve the individual's connection with everyday life, and provide a more natural transition for subsequent recovery. This principle is in line with a functional rehabilitation approach, as it does not prematurely shift the individual into the role of a patient, but instead maintains the connection between the individual and the familiar environment, daily roles, and practical actions. BICEPS provides a set of operational principles for early support rather than a standardized psychotherapeutic protocol. Its clinical value lies in guiding brief, proximal, simple, and function-oriented support, but its independent effect size has not been clearly established in controlled studies.

During the 24- to 72-h window after trauma, the focus of early support should not be limited to reassurance, but should instead center on targeted observation and stratification of functional recovery. Specifically, attention should be paid to whether sleep and eating gradually improve, whether the individual is able to perform self-care and make simple judgments, whether persistent hypervigilance, avoidance, panic, or recurrent recollections continue to impair work, cooperation with medical care, and social interaction, and whether family, peer, or organizational support resources are available (Schnurr et al., 2024; Lang et al., 2024; Card, 2017). These indicators help operationalize recovery in concrete bodily, behavioral, and functional terms. If, within a supportive environment, the individual gradually regains basic daily rhythms, emotional fluctuations decrease, and real-world functioning improves, ongoing observation and support may be continued. If symptoms remain present but are generally manageable, short-term follow-up and supportive care should be intensified to reduce the risk of chronicity. By contrast, if symptoms worsen further, functioning deteriorates, or risk factors such as impulsivity, self-harm, suicidal ideation, severe behavioral disturbance, or exacerbation of pre-existing mental disorders emerge, the individual should be transitioned to the next stage of crisis intervention or specialist assessment (Lang et al., 2024).

### 5.3 Structured early treatment for high-risk individuals

The crisis intervention model is mainly intended for two groups: individuals who display clear high-risk warning signs and those who have received early intervention but shown a poor response (Tripathi et al., 2023). Compared with general function-oriented supportive frameworks, the primary role of crisis intervention is to control risk, address immediate problems, and guide short-term decision-making. According to this model, target populations include individuals with severe emotional disturbance, increased risk of impulsive behavior, and worsening mental health status (Tripathi et al., 2023; Ghelani, 2022). In practice, the first phase of crisis intervention focuses on problem identification, safety assurance, and support provision, thereby laying the foundation for subsequent action. The second phase emphasizes evaluation of available options, development of a response plan, and obtaining the individual's commitment to implementation. This model requires clinicians to help individuals rapidly clarify the core crisis, reduce immediate danger, and formulate short-term, actionable coping measures; when necessary, emergency services, specialist mental health care, or crisis response systems should be engaged immediately. During the early intervention stage, crisis intervention should not replace routine supportive care; rather, it functions as a critical step in transitioning from supportive observation to specialist management when risk escalates. In this framework, IMPRESS and BICEPS provide largely principle-based and practice-oriented guidance for restoring function, reducing fixation in the sick role, and maintaining connection with everyday life, whereas crisis intervention represents an escalated strategy for individuals whose symptoms, behavior, or functional impairment indicate immediate risk. Effective support at this stage can maximize natural recovery, reduce symptom persistence, and provide a stratified foundation for subsequent structured treatment of high-risk patients.

Trauma-focused cognitive behavioral therapy (TF-CBT) is one of the most representative structured treatments for high-risk individuals with persistent acute traumatic stress symptoms. It focuses on traumatic memories and associated negative cognitions, and employs modular techniques such as cognitive restructuring, anxiety management, behavioral activation, and exposure to help patients modify maladaptive trauma-related appraisals, reduce avoidance, regulate arousal, and gradually restore everyday functioning (Schnurr et al., 2024; Lang et al., 2024; Kalla et al., 2024). For adults with acute traumatic stress symptoms accompanied by significant functional impairment, current recommendations suggest that TF-CBT may be considered (Kalla et al., 2024). A Cochrane review of early psychological interventions for acute traumatic stress symptoms identified 15 studies, including 12 studies evaluating brief TF-CBT. In pooled analyses, TF-CBT was more effective than wait-list control in reducing acute traumatic stress symptoms at initial follow-up (six studies, 471 participants; SMD = −0.64, 95% CI: −1.06 to −0.23), and more effective than supportive counseling (4 studies, 198 participants; SMD = −0.67, 95% CI: −1.12 to −0.23). The benefit over supportive counseling remained at 6-month follow-up (four studies, 170 participants; SMD = −0.64, 95% CI: −1.02 to −0.25). TF-CBT was also associated with short-term reductions in anxiety (seven studies, 513 participants; SMD = −0.53, 95% CI: −0.91 to −0.15) and depressive symptoms (seven studies, 513 participants; SMD = −0.49, 95% CI: −0.80 to −0.18) (Roberts et al., 2010).

Within the staged management of ASR, TF-CBT should not be regarded as a universal preventive strategy for all trauma-exposed individuals, but rather as an early treatment approach with specific indications. Suitable candidates are those who have developed persistent symptoms, marked avoidance, ongoing hyperarousal, or functional impairment and who retain sufficient emotional tolerance and treatment engagement to participate in trauma-focused work (Roberts et al., 2019). In practice, TF-CBT may be delivered through brief, repeated, structured sessions focused primarily on trauma memory processing, reduction of avoidance, modulation of arousal and restoration of real-world functioning. At the same time, this form of treatment places greater demands on adherence, therapist expertise, and the individual's capacity to tolerate emotional activation during therapy. Accordingly, precise screening and stratified application should be considered the fundamental principles guiding its use (Roberts et al., 2019).

Eye movement desensitization and reprocessing (EMDR) may be considered as an early treatment option for selected high-risk individuals, particularly those with persistent post-traumatic symptoms or functional impairment after initial screening and assessment. Recommendations regarding its use within the first 3 months after trauma remain inconsistent across authoritative guidelines (Wright et al., 2024). Some guidelines take a favorable view and suggest that EMDR may be incorporated into early treatment plans for adults with post-traumatic stress symptoms (National Institute for Health Care Excellence, 2018). Group-based EMDR protocols, including G-TEP, R-TEP, and EMDR Flash Technique, have been applied in mass-trauma contexts such as natural disasters, forced displacement, and pandemic-related occupational trauma. Evidence from Syrian refugees, frontline or emergency workers during the COVID-19 pandemic suggests that early group-based EMDR may be feasible and potentially beneficial in selected populations, but the strongest supporting evidence is still derived mainly from studies conducted at longer intervals after trauma (Torres-Giménez et al., 2024; Yurtsever et al., 2018; Farrell et al., 2023). Because the evidence remains heterogeneous, the decision to use EMDR within the first 1 to 3 months after trauma should be based on symptom severity, functional impairment, patient preference, treatment availability, and the applicability of the available evidence (Torres-Giménez et al., 2024). EMDR is better regarded as a selective early treatment option for individuals with persistent symptoms rather than as a routine or universal intervention for all trauma-exposed individuals.

During the period from several days to several weeks after trauma, early treatment for high-risk individuals should be based on prior assessment and symptom stratification, with targeted intervention implemented only after precise identification, rather than through a generalized and uniform approach applied to all trauma-exposed individuals. Within the current body of evidence, TF-CBT remains the most representative structured early treatment and is particularly suitable for individuals with persistent symptoms, marked avoidance or hyperarousal, and clearly significant functional impairment. EMDR may be considered as a selective treatment option; however, its use in the early post-trauma period requires careful attention to the scope and applicability of the available evidence, and clinical decisions should be made cautiously in light of the individual's specific condition. Brief narrative exposure therapy may have translational potential in resource-limited settings, but the evidence base for its early use after trauma remains more limited and should be interpreted cautiously. Structured early treatment should be proportionate, stratified, and function-oriented, aiming to reduce symptom severity, restore emotional regulation, behavioral engagement, and everyday functioning. This approach may help reduce the individual's symptom persistence and facilitate a transition in time-sensitive ASR intervention from supportive stabilization to precision-oriented treatment.

### 5.4 Supplementary measures: medication, mindfulness, and social support

The use of pharmacological intervention is subject to strict limitations and should be considered only as a short-term adjunctive measure for specific symptoms. When individuals experience severe insomnia, intense anxiety, or marked physiological hyperarousal during the early post-traumatic period, medication may be used selectively to relieve acute distress and support short-term stabilization. If the patient also presents with comorbid major depression, psychotic symptoms, or other clearly defined psychiatric disorders, pharmacotherapy may be incorporated into a comprehensive treatment plan. A Cochrane review of early pharmacological interventions included 13 studies involving 2,023 participants and evaluated hydrocortisone, propranolol, dexamethasone, omega-3 fatty acids, gabapentin, paroxetine, enteral formulas, and 5-hydroxytryptophan. The review concluded that evidence for these medications as universal PTSD prevention strategies was uncertain, and noted important gaps in adverse-event reporting, functional disability, and quality-of-life outcomes (Bertolini et al., 2022).

Regarding the use of medication for the prevention of PTSD, current mainstream guidelines generally adopt a cautious or even negative stance (Schnurr et al., 2024). In particular, routine use of benzodiazepines is discouraged. Benzodiazepines have not shown reliable preventive benefit for PTSD and may even be associated with increased subsequent PTSD risk (Schnurr et al., 2024). A systematic review and meta-analysis reported a higher risk of PTSD among benzodiazepine recipients after potentially traumatic events (Campos et al., 2022). By contrast, propranolol has a more mechanistically plausible but empirically inconsistent profile. Beta-adrenergic blockade during the early memory-consolidation window may theoretically attenuate the emotional salience of traumatic memories (Pitman and Delahanty, 2005); however, randomized prevention trials have produced negative or only limited findings, and recent meta-analytic evidence suggests only preliminary symptom-level benefit, with unresolved questions regarding timing, dosage, target population, and long-term safety (Stein et al., 2007; Hoge et al., 2012; Li et al., 2025). In time-sensitive intervention for ASR, medication is therefore better regarded as a short-term supportive measure for specific symptoms or comorbid problems, rather than as a routine preventive intervention.

Mindfulness-based techniques in the management of ASR should be regarded as minimally intrusive adjunctive approaches for stabilization, bodily awareness, and self-regulation. Mindfulness-based stress reduction, mindfulness-based cognitive therapy, and other integrative mindfulness training programs generally aim to reduce persistent emotional and physiological arousal triggered by automatic negative thoughts and stress-response patterns through attentional guidance, body awareness, breathing regulation, mindfulness practice, and acceptance-based exercises, while enhancing the individual's capacity to regulate stress-related experiences (Li et al., 2024). Meta-analytic and umbrella-review evidence suggests that mindfulness-based interventions may reduce PTSD symptoms, but treatment effects vary across studies and these interventions are still used primarily as adjunctive therapeutic approaches rather than as stand-alone early trauma treatments (Liu Q. et al., 2022; Jovanovic and Garfin, 2024). During the acute period spanning several days to several weeks after trauma, brief and practical mindfulness techniques, such as breath anchoring, body scanning, and grounding skills, should be preferred. These methods may support early psychological care by improving sleep patterns, reducing physiological arousal, strengthening awareness of bodily sensations, and helping affected individuals regain basic daily rhythms (Powers et al., 2023). It should be noted, however, that for individuals with marked dissociative symptoms, frequent intrusive traumatic memories, or prominent avoidance behaviors, mindfulness training alone is usually insufficient to address the main clinical problems. In such cases, trauma-focused therapy should be initiated promptly or referral to specialist care should be arranged.

After trauma exposure, the social support available to an individual during recovery constitutes a relatively stable and long-term protective factor that may influence both psychological adjustment and functional recovery. It not only helps the individual withstand the initial psychological impact, but also facilitates later adaptation to daily life, restoration of social roles, and recovery of physical functioning (Calhoun et al., 2022). Higher perceived social support is generally associated with milder post-traumatic stress symptoms and better recovery of daily functioning. Current evidence further suggests a possible bidirectional interaction between these two factors: the more severe the symptoms, the more likely the individual is to gradually withdraw from social relationships and become less receptive to support; conversely, the weaker the support system, the more limited the individual's access to resources and capacity to seek help actively during recovery (Wang et al., 2021; Liu S. Y. et al., 2022).

In time-sensitive intervention for ASR, social support is not a peripheral factor, but a core component that runs through the entire course of care and helps sustain recovery in real-world contexts. According to the form of intervention, social support can be broadly categorized into three types. The first consists of connection and referral, the core purpose of which is to help individuals rapidly access existing resources, including relatives or friends, medical services, practical assistance with daily living, and other channels of social support that can stabilize everyday functioning (Wang et al., 2024). The implementation of this type of support closely parallels the “link” component of PFA and can reduce isolation, alleviate anxiety, and help restore short-term social functioning (Hermosilla et al., 2023). The second type involves collaborative support provided by peers and organizations, and is more commonly applied in high-risk occupational groups such as firefighters, healthcare workers, military personnel, and police officers. This form of support can strengthen peer connectedness, improve the internal organizational climate, reduce the sense of isolation and stigma associated with ASR, and enable individuals to maintain behavioral engagement and cope more sustainably in high-pressure environments (Fallon et al., 2023). The third type is empowerment-based support directed toward families and significant others, primarily through training for family members and key supporters. Such training may improve their ability to identify warning signs accurately, communicate more effectively, and provide practical assistance for sleep, daily routines, help-seeking, and safety planning, thereby enhancing the overall functioning of the individual's support system (Meuleman et al., 2023; Thompson-Hollands et al., 2021). Although these three forms of social support differ in their specific modes of delivery, their shared purpose is to reduce the individual's sense of isolation, improve access to external resources, and facilitate active help-seeking and sustained adaptation throughout recovery.

The use of supplementary measures should always follow the principle of stepped intervention (Schnurr et al., 2024). For most individuals with mild to moderate ASR who do not show high-risk behavioral indicators, mindfulness-informed techniques and structured social support may serve as non-pharmacological adjunctive strategies to improve sleep, reduce hyperarousal, enhance a sense of safety, and maintain basic functioning. Pharmacotherapy should be reserved for selected cases with severe insomnia, severe anxiety, marked physiological hyperarousal, or clearly defined comorbid psychiatric conditions, rather than used as a routine preventive or supportive intervention. Supplementary measures are best applied not as stand-alone interventions, but as optional components integrated into a comprehensive and function-oriented system of assessment, stratification, intervention, and referral tailored to the individual's symptoms, bodily regulation, functional status, and clinical risk profile (Zatzick et al., 2021).

## 6 Discussion

**Figure 1:**
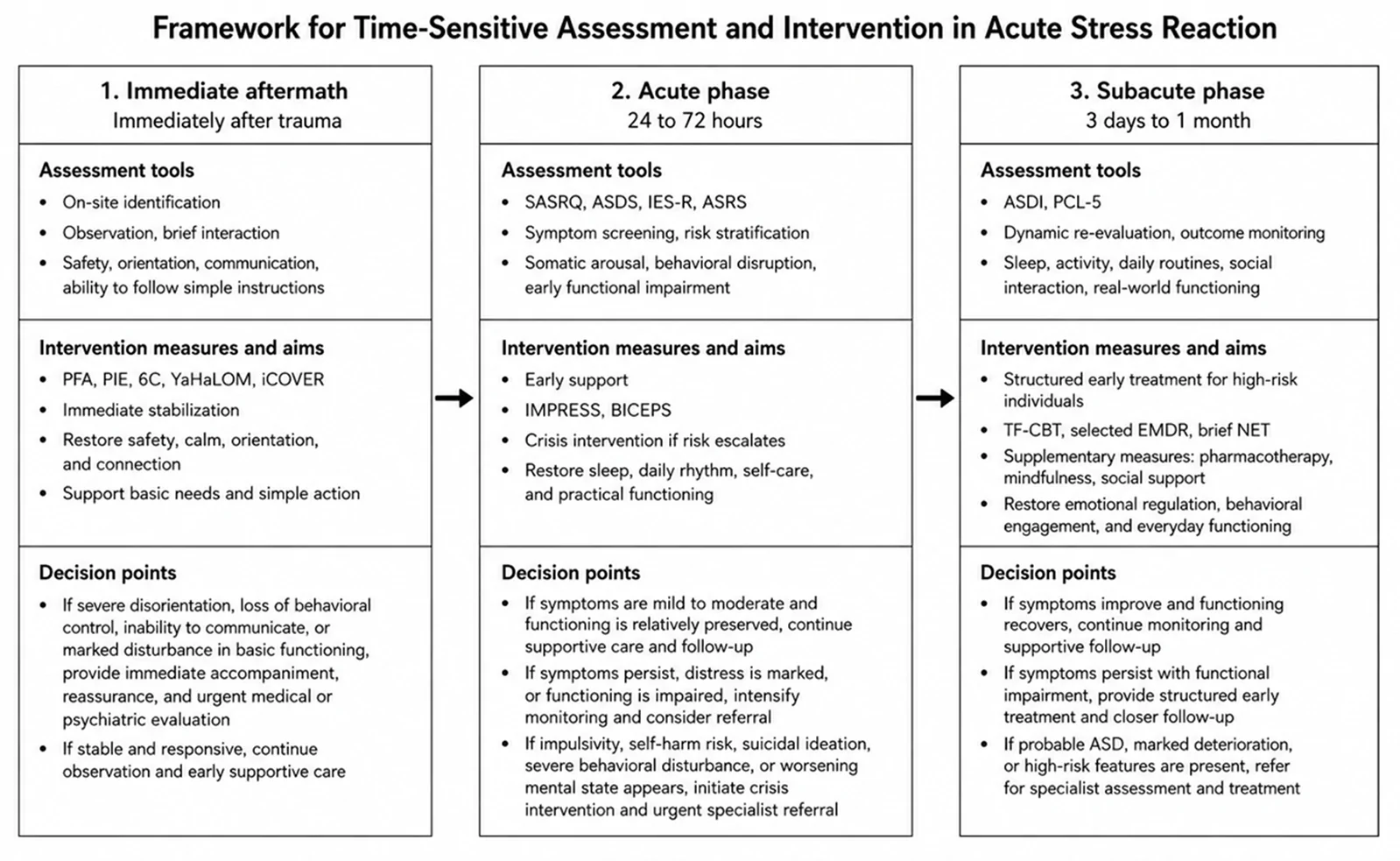
Framework for time-sensitive assessment and intervention in acute stress reaction.

The main contribution of this review is to organize early post-traumatic assessment and support into a staged, time-sensitive framework linking the immediate, acute, and subacute windows with symptom screening, somatic and functional indicators, risk stratification, and referral decision-making. To visually present this approach, Figure 1 links assessment tools, intervention aims, and decision points within a time-sensitive model of ASR management. Within this framework, the embodied dimension of ASR is conceptualized in a clinically operational manner. Acute stress responses are understood as patterns that involve subjective distress, cognitive appraisal, interoceptive discomfort, autonomic arousal, sensorimotor or behavioral organization, physiological stress-system activity, and disruption of everyday functioning. These variables can be operationalized at multiple levels: brief clinical observation of posture, agitation, freezing, communication, and action readiness in the immediate phase; self-reported somatic arousal, dissociation, sleep disturbance, and functional impairment during acute and subacute assessment; and adjunctive physiological or digital indicators such as HRV, cortisol/HPA-axis activity, wearable-derived sleep and activity data, EMA, and digital phenotyping for dynamic monitoring. These indicators are not treated as stand-alone diagnostic markers, but as complementary sources of information that may help clinicians understand bodily regulation, functional recovery, and changes in perceived safety or threat over time. This framework is primarily intended for adults who can participate in brief clinical interaction and self-report-based symptom assessment. For children and adolescents, older adults, individuals with cognitive or communication impairment, and cultural or linguistic minorities, the framework should be applied cautiously and adapted through developmentally appropriate assessment, collateral information, interpreter or caregiver involvement, and clinician judgment.

The core goal of time-sensitive intervention for ASR include short-term symptom relief, restoration of bodily regulation, behavioral engagement, and everyday functioning. Across different intervention approaches, a common therapeutic aim is the restoration of essential bodily and functional capacities, including the ability to sleep, eat, move, communicate, and return to daily life. Some individuals may show prominent ASR-related symptoms early on but recover function rapidly, whereas others may have less obvious symptoms yet continue to experience persistent functional impairment in real-world settings. Symptom severity, bodily regulation, and functional status should therefore not be treated as separate dimensions. Instead, longitudinal observation and reassessment are needed to determine whether natural recovery is progressing, whether embodied and functional recovery has stalled, and whether more structured treatment or crisis intervention is warranted.

From this embodied and clinically operational perspective, ASR assessment should integrate bodily sensations, observable behavior, physiological arousal, and functional status with symptom-based evaluation. These indicators include self-reported somatic discomfort and hyperarousal; observable behavioral signs, such as immobility, agitation, or disorientation; functional indicators, including sleep, activity, and daily routines; and physiological or digital markers, such as heart rate variability, skin conductance, passive sensing, and ecological momentary assessment. Together, these indicators capture the dynamic and context-dependent nature of ASR and can complement traditional symptom-based scales.

Existing evidence still shows substantial imbalance in both research settings and population distribution. The theoretical development and innovation of intervention programs for ASR have been heavily shaped by military contexts, disaster rescue teams, and other high-pressure operational environments. Accordingly, typical immediate intervention models such as PIE, 6C, YaHaLOM, and iCOVER were primarily developed in real combat or combat-analogous settings and validated within systems characterized by strong organizational discipline, close peer relationships, and clearly defined task goals. Although these studies provide important insights into the early management of ASR, their implementation requirements, organizational culture, and operational goals and target populations cannot be fully transferred to civilian healthcare, emergency services, or general trauma settings. Uncritical direct application may result in a mismatch between intervention goals and implementation conditions, thereby producing inconsistent outcomes. Research in hospital emergency departments, routine clinical services, and populations frequently exposed to traumatic environments remains relatively limited. In particular, there is a lack of process validation and effectiveness evaluation conducted across different healthcare systems and national contexts.

In addition, most studies rely on short-term symptom improvement as the primary outcome, whereas relatively few report long-term prognosis or recovery of real-world functioning. Some studies evaluate feasibility and effectiveness using indicators such as score reduction, post-training knowledge acquisition, or completion rates of procedural steps during role-play exercises. However, these measures cannot fully determine whether an intervention effectively promotes functional recovery, reduces the persistence or worsening of ASR-related symptoms, lowers subsequent PTSD risk, or improves subsequent referral pathways and continuity of care. For ASR, outcomes of genuine clinical relevance should extend beyond symptom relief to include sleep, nutrition, activity, communication, cooperation, social functioning, and even the incidence of adverse outcomes in the weeks or months after trauma.

Current research also has important methodological limitations. Some studies have included only small samples, with participants drawn predominantly from a single institution or unit. Consequently, their findings often represent experience derived from a particular team, country, or trauma type and therefore have limited generalizability. In addition, some early intervention approaches cannot readily be evaluated in rigorous randomized controlled trials (RCTs) because of ethical constraints, field conditions, and insufficient follow-up, which lowers the overall certainty of the evidence. At the same time, studies differ in how they define ASR, ASD, and post-traumatic stress disorder. Assessment timing, inclusion criteria, and outcome measures are also not fully standardized, making cross-study comparison and integration difficult.

This review and the evidence on which it is based have some limitations. This review included a staged comparison of commonly used assessment tools and intervention models, but no formal comparative effectiveness or cost-benefit analyses were performed. Thus, it is not possible to determine whether one tool or model is superior to another. The existing literature is still insufficient to explore the implementation barriers. The practical application of time-sensitive ASR assessments and interventions may be constrained by personnel training, time pressures in emergency or disaster settings, institutional referral processes, cultural adaptations, stigma of psychological care, and access to specialized mental health services. The framework presented here may need to be adapted for special populations, including children and adolescents, older adults, individuals with preexisting mental health problems, cultural minorities, and patients with communication or cognitive impairments. These populations may differ in terms of symptom presentation, communication skills, family or caregiver engagement, the effectiveness of assessment tools, and the tolerance of trauma-focused interventions.

Future research should focus on continuous and dynamic assessment and intervention across different post-trauma periods, with the aim of developing time-sensitive care approaches for ASR that integrate dynamic assessment, symptom quantification and stratification, symptom-targeted intervention, and outcome monitoring. Although assessment approaches are now relatively well developed, intervention strategies still require further validation or refinement in real-world clinical and high-pressure settings. Such refinement should take into account core elements such as intervention timing and intensity, while incorporating outcome measures related to functional recovery and prognosis. Future research may also further develop monitoring models based on ecological momentary assessment, passive sensing technologies, and multimodal data fusion to enable high-frequency longitudinal tracking and early risk warning, but caution is needed to avoid overinterpretation of any single indicator. Through these efforts, it may be possible to further improve both the generalizability and the evidence base of time-sensitive care systems for ASR.

## References

[B1] Adler A. B. Gutierrez I. A. (2022a). Acute stress reaction in combat: emerging evidence and peer-based interventions. Curr. Psychiatry Rep. 24, 277–284. doi: 10.1007/s11920-022-01335-2 35353322 PMC8965216

[B2] Adler A. B. Gutierrez I. A. (2022b). Preparing soldiers to manage acute stress in combat: acceptability, knowledge and attitudes. Psychiatry 85, 30–37. doi: 10.1080/00332747.2021.2021598 35138988

[B3] Adler A. B. Gutierrez I. A. McCuaig Edge H. Nordstrand A. E. Simms A. Willmund G. D. . (2024). Peer-based intervention for acute stress reaction: adaptations by five militaries. BMJ Mil. Health 170, 425–429. doi: 10.1136/military-2022-002344 37280014 PMC11503197

[B4] Adler A. B. Start A. R. Milham L. Allard Y. S. Riddle D. Townsend L. . (2020). Rapid response to acute stress reaction: pilot test of iCOVER training for military units. Psychol. Trauma 12, 431–435. doi: 10.1037/tra0000487 31328939

[B5] American Psychiatric Association (2022). Diagnostic and Statistical Manual of Mental Disorders. Washington, DC: American Psychiatric Association Publishing. doi: 10.1176/appi.books.9780890425787

[B6] Belenky G. L. Tyner C. F. Sodetz F. J. (1983). Israeli Battle Shock Casualties: 1973 and 1982. Washington, DC: Division of Neuropsychiatry, Walter Reed Army Institute of Research.

[B7] Bertolini F. Robertson L. Bisson J. I. Meader N. Churchill R. Ostuzzi G. . (2022). Early pharmacological interventions for universal prevention of post-traumatic stress disorder (PTSD). Cochrane Database Syst. Rev. 2:Cd013443. doi: 10.1002/14651858.CD013443.pub2 PMC8829470 35141873

[B8] Blevins C. A. Weathers F. W. Davis M. T. Witte T. K. Domino J. L. (2015). The posttraumatic stress disorder checklist for DSM-5 (PCL-5): development and initial psychometric evaluation. J. Trauma Stress 28, 489–498. doi: 10.1002/jts.22059 26606250

[B9] Blood C. G. Gauker E. D. (1993). The relationship between battle intensity and disease rates among marine corps infantry units. Mil. Med. 158, 340–344. doi: 10.1093/milmed/158.5.340 8502400

[B10] Bovin M. J. Marx B. P. (2023). The problem with overreliance on the PCL−5 as a measure of PTSD diagnostic status. Clin. Psychol. 30, 122–125. doi: 10.1037/cps0000119

[B11] Brier Z. M. F. Connor J. Legrand A. C. Price M. (2020). Different trajectories of PTSD symptoms during the acute post-trauma period. J. Psychiatr. Res. 131, 127–131. doi: 10.1016/j.jpsychires.2020.08.037 32961502 PMC7676876

[B12] Brier Z. M. F. Hidalgo J. E. Espeleta H. C. Davidson T. Ruggiero K. J. Price M. . (2023). Assessment of traumatic stress symptoms during the acute posttrauma period. Focus 21, 239–246. doi: 10.1176/appi.focus.20230001 37404969 PMC10316216

[B13] Bryant R. Harvey A. Dang S. Sackville T. (1998). Assessing acute stress disorder: psychometric properties of a structured clinical interview. Psychol. Assess. 10, 215–220. doi: 10.1037/1040-3590.10.3.215

[B14] Bryant R. A. Moulds M. L. Guthrie R. M. (2000). Acute stress disorder scale: a self-report measure of acute stress disorder. Psychol. Assess. 12, 61–68. doi: 10.1037/1040-3590.12.1.61 10752364

[B15] Cadeddu R. Braccagni G. Floris G. Branca C. Corridori E. Salviati S. . (2025). Prefrontal 5α-reductase 2 mediates male-specific acute stress response. Sci. Adv. 11:eadr0563. doi: 10.1126/sciadv.adr0563 39841836 PMC11753402

[B16] Calhoun C. D. Stone K. J. Cobb A. R. Patterson M. W. Danielson C. K. Bendezú J. J. . (2022). The role of social support in coping with psychological trauma: an integrated biopsychosocial model for posttraumatic stress recovery. Psychiatr. Q. 93, 949–970. doi: 10.1007/s11126-022-10003-w 36199000 PMC9534006

[B17] Campos B. Vinder V. Passos R. B. F. Coutinho E. S. F. Vieira N. C. P. Leal K. B. . (2022). To BDZ or not to BDZ? That is the question! Is there reliable scientific evidence for or against using benzodiazepines in the aftermath of potentially traumatic events for the prevention of PTSD? A systematic review and meta-analysis. J. Psychopharmacol. 36, 449–459. doi: 10.1177/02698811221080464 35437077

[B18] Cancio J. M. Cancio L. C. (2023). Combat and operational stress control: application in a burn center. Eur. Burn. J. 5, 12–22. doi: 10.3390/ebj5010002 39600010 PMC11571823

[B19] Card P. (2017). VA/DOD clinical practice guideline for the management of posttraumatic stress disorder and acute stress disorder. Focus 16, 430–448. doi: 10.1176/appi.focus.16408 32021581 PMC6996084

[B20] Cardeña E. Koopman C. Classen C. Waelde L. C. Spiegel D. (2000). Psychometric properties of the stanford acute stress reaction questionnaire (SASRQ): a valid and reliable measure of acute stress. J. Trauma. Stress 13, 719–734. doi: 10.1023/A:1007822603186 11109242

[B21] Cooke L. C. E. T. (2014). “Combat stress and its effects: combat's bloodless casualties,” in US Army Psychiatry in the Vietnam War: New Challenges in Extended Counterinsurgency Warfare, eds. D. E. Banks, and L. Sparacino (Fort Sam Houston, TX: Borden Institute), 145.

[B22] Cooper D. C. Campbell M. S. Baisley M. Hein C. L. Hoyt T. (2024). Combat and operational stress programs and interventions: a scoping review using a tiered prevention framework. Mil. Psychol. 36, 253–265. doi: 10.1080/08995605.2021.1968289 38661468 PMC11057676

[B23] Dai W. Liu A. Kaminga A. C. Deng J. Lai Z. Yang J. . (2018). Prevalence of acute stress disorder among road traffic accident survivors: a meta-analysis. BMC Psychiatry 18:188. doi: 10.1186/s12888-018-1769-9 29895273 PMC5998549

[B24] Dutheil F. de Saint Vincent S. Pereira B. Schmidt J. Moustafa F. Charkhabi M. . (2021). DHEA as a biomarker of stress: a systematic review and meta-analysis. Front. Psychiatry 12:688367. doi: 10.3389/fpsyt.2021.688367 34295276 PMC8290065

[B25] Fallon P. Jaegers L. A. Zhang Y. Dugan A. G. Cherniack M. El Ghaziri M. . (2023). Peer support programs to reduce organizational stress and trauma for public safety workers: a scoping review. Workplace Health Saf. 71, 523–535. doi: 10.1177/21650799231194623 37702192

[B26] Farchi M. Levy T. B. Gershon B. B. Hirsch-Gornemann M. B. Whiteson A. Gidron Y. . (2018). The six CS model for immediate cognitive psychological first aid: from helplessness to active efficient coping. Int. J. Emerg. Ment. Health Hum. Resil. 20, 1–12. doi: 10.4172/1522-4821.1000395

[B27] Farchi M. U. Bathish L. Hayut N. Alexander S. Gidron Y. (2025). Effects of a psychological first aid (PFA) based on the six CS model on acute stress responses in a simulated emergency. Psychol. Trauma 17, 897–903. doi: 10.1037/tra0001724 39052417

[B28] Farrell D. Moran J. Zat Z. Miller P. W. Knibbs L. Papanikolopoulos P. . (2023). Group early intervention eye movement desensitization and reprocessing therapy as a video-conference psychotherapy with frontline/emergency workers in response to the COVID-19 pandemic in the treatment of post-traumatic stress disorder and moral injury-an RCT study. Front. Psychol. 14:1129912. doi: 10.3389/fpsyg.2023.1129912 37063579 PMC10100089

[B29] Forkus S. R. Raudales A. M. Rafiuddin H. S. Weiss N. H. Messman B. A. Contractor A. A. . (2023). The posttraumatic stress disorder (PTSD) checklist for DSM-5: a systematic review of existing psychometric evidence. Clin. Psychol. 30, 110–121. doi: 10.1037/cps0000111 37378352 PMC10292741

[B30] Garcia-Esteve L. Torres-Gimenez A. Canto M. Roca-Lecumberri A. Roda E. Velasco E. R. . (2021). Prevalence and risk factors for acute stress disorder in female victims of sexual assault. Psychiatry Res. 306:114240. doi: 10.1016/j.psychres.2021.114240 34673311

[B31] Ghelani A. (2022). Knowledge and skills for social workers on mobile crisis intervention teams. Clin. Soc. Work J. 50, 414–425. doi: 10.1007/s10615-021-00823-x 34803191 PMC8591592

[B32] Gong Y. Guo Z. Lu H. Wang X. Zhang Y. Ren L. . (2023). Network analysis of acute stress reaction in a sample of Chinese male military college students. Front. Psychiatry 14:1082549. doi: 10.3389/fpsyt.2023.1082549 37621968 PMC10444979

[B33] Hagos T. G. Tamir T. T. Workneh B. S. Abrha N. N. Demissie N. G. Gebeyehu D. A. . (2024). Acute stress disorder and associated factors among adult trauma patients in Ethiopia: a multi-institutional study. BMC Psychiatry 24:418. doi: 10.1186/s12888-024-05861-6 38834988 PMC11151476

[B34] Helmus T. C. Glenn R. W. (2005). Steeling the Mind: Combat Stress Reactions and Their Implications for Urban Warfare. Santa Monica, CA: RAND Corporation. doi: 10.1037/e426122005-001

[B35] Hermosilla S. Forthal S. Sadowska K. Magill E. B. Watson P. Pike K. M. . (2023). We need to build the evidence: a systematic review of psychological first aid on mental health and well-being. J. Trauma Stress 36, 5–16. doi: 10.1002/jts.22888 36300605 PMC10624106

[B36] Hoge E. A. Worthington J. J. Nagurney J. T. Chang Y. Kay E. B. Feterowski C. M. . (2012). Effect of acute posttrauma propranolol on PTSD outcome and physiological responses during script-driven imagery. CNS Neurosci. Ther. 18, 21–27. doi: 10.1111/j.1755-5949.2010.00227.x 22070357 PMC6493400

[B37] Hruska B. Piccirillo M. L. Lenferink L. I. M. Pacella-LaBarbara M. L. Contractor A. A. Price M. . (2025). Making trauma ecological momentary assessment studies fair: review of design considerations and data procedures. Eur. J. Psychotraumatol. 16:2477423. doi: 10.1080/20008066.2025.2477423 40116183 PMC11934184

[B38] Jensen S. M. Abrahamsen I. Baumgarten M. Gallaher J. Feltner C. (2022). Screening tools for predicting posttraumatic stress disorder in acutely injured adult trauma patients: a systematic review. J. Trauma Acute Care Surg. 92, e115–e126. doi: 10.1097/TA.0000000000003524 34991124

[B39] Jones E. Wessely S. (2001). Psychiatric battle casualties: an intra-and interwar comparison. Br. J. Psychiatry 178, 242–247. doi: 10.1192/bjp.178.3.242 11230035

[B40] Jovanovic B. Garfin D. R. (2024). Can mindfulness-based interventions reduce PTSD symptoms? An Umbrella Review. J. Anxiety Disord. 104:102859. doi: 10.1016/j.janxdis.2024.102859 38761551 PMC12092160

[B41] Kalla C. Lifshitz M. Shelef L. Tatsa-Laur L. Fruchter E. Lotan A. . (2024). [Therapeutic interventions in acute stress disorder (ASD)]. Harefuah 163, 640–644. 39535013

[B42] Kessler R. C. Ressler K. J. House S. L. Beaudoin F. L. An X. Stevens J. S. . (2021). Socio-demographic and trauma-related predictors of PTSD within 8 weeks of a motor vehicle collision in the Aurora study. Mol. Psychiatry 26, 3108–3121. doi: 10.1038/s41380-020-00911-3 33077855 PMC8053721

[B43] Kim E. Y. Han S. W. (2021). Development of psychological first aid guidelines for people who have experienced disasters. Int. J. Environ. Res. Public Health 18:10752. doi: 10.3390/ijerph182010752 34682491 PMC8535812

[B44] Kramer L. B. Whiteman S. E. Petri J. M. Spitzer E. G. Weathers F. W. (2023). Self-rated versus clinician-rated assessment of posttraumatic stress disorder: an evaluation of discrepancies between the PTSD checklist for DSM-5 and the clinician-administered PTSD scale for DSM-5. Assessment 30, 1590–1605. doi: 10.1177/10731911221113571 35915927

[B45] Krupelnytska L. Yatsenko N. Keller V. Morozova-Larina O. (2025). The impact of events scale-revised (IES-R): validation of the Ukrainian version. Compr. Psychiatry 139:152593. doi: 10.1016/j.comppsych.2025.152593 40168846

[B46] Lang A. J. Hamblen J. L. Holtzheimer P. Kelly U. Norman S. B. Riggs D. . (2024). A clinician's guide to the 2023 VA/DOD clinical practice guideline for management of posttraumatic stress disorder and acute stress disorder. J. Trauma Stress 37, 19–34. doi: 10.1002/jts.23013 38184799

[B47] Li H. Zhang Z. Yang S. Zhu G. (2025). Systematic review and meta-analysis of propranolol in the prevention and treatment of post-traumatic stress disorder. Front. Pharmacol. 16:1545493. doi: 10.3389/fphar.2025.1545493 39944616 PMC11814184

[B48] Li W. W. Nannestad J. Leow T. Heward C. (2024). The effectiveness of mindfulness-based stress reduction (MBSR) on depression, PTSD, and mindfulness among military veterans: a systematic review and meta-analysis. Health Psychol. Open 11:20551029241302969. doi: 10.1177/20551029241302969 39582518 PMC11583271

[B49] Liu Q. Zhu J. Zhang W. (2022). The efficacy of mindfulness-based stress reduction intervention for post-traumatic stress disorder (PTSD) symptoms in patients with PTSD: a meta-analysis of four randomized controlled trials. Stress Health 38, 626–636. doi: 10.1002/smi.3138 35253353

[B50] Liu S. Y. Li J. Leon L. F. Schwarzer R. Cone J. E. (2022). The bidirectional relationship between posttraumatic stress symptoms and social support in a 9/11-exposed cohort: a longitudinal cross-lagged analysis. Int. J. Environ. Res. Public Health 19:2604. doi: 10.3390/ijerph19052604 35270297 PMC8910094

[B51] Lötvall R. Palmborg Å. Cardeña E. (2022). A 20-years+ review of the stanford acute stress reaction questionnaire (SASRQ): psychometric properties and findings. Eur. J. Trauma Dissoc. 6:100269. doi: 10.1016/j.ejtd.2022.100269

[B52] Matson L. M. Adler A. B. Quartana P. J. Thomas C. L. Lowery-Gionta E. G. (2022). Management of acute stress reactions in the military: a stepped care approach. Curr. Psychiatry Rep. 24, 799–808. doi: 10.1007/s11920-022-01388-3 36538195 PMC9780143

[B53] Meuleman E. Sloover M. van Ee E. (2023). Involving a significant other in treatment of patients with PTSD symptoms: a systematic review of treatment interventions. Trauma Violence Abuse 24, 2034–2044. doi: 10.1177/15248380221082939 35389279

[B54] Morris M. C. Hellman N. Abelson J. L. Rao U. (2016). Cortisol, heart rate, and blood pressure as early markers of PTSD risk: a systematic review and meta-analysis. Clin. Psychol. Rev. 49, 79–91. doi: 10.1016/j.cpr.2016.09.001 27623149 PMC5079809

[B55] Mouthaan J. Sijbrandij M. Luitse J. S. Goslings J. C. Gersons B. P. Olff M. . (2014). The role of acute cortisol and DHEAS in predicting acute and chronic PTSD symptoms. Psychoneuroendocrinology 45, 179–186. doi: 10.1016/j.psyneuen.2014.04.001 24845188

[B56] National Institute for Health and Care Excellence (2018). Post-Traumatic Stress Disorder. NICE Guideline NG116. London: National Institute for Health and Care Excellence.

[B57] O'Connor K. E. Shanholtz C. E. Espeleta H. C. Ridings L. E. Gavrilova Y. Hink A. . (2024). Mental health symptoms and engagement in a stepped-care mental health service among patients with a violent versus nonviolent injury. J. Trauma Acute Care Surg. 96, 650–657. doi: 10.1097/TA.0000000000004078 37339343 PMC10733549

[B58] Pinge A. Gad V. Jaisighani D. Ghosh S. Sen S. (2024). Detection and monitoring of stress using wearables: a systematic review. Front. Comput. Sci. 6:1478851. doi: 10.3389/fcomp.2024.1478851

[B59] Pitman R. K. Delahanty D. L. (2005). Conceptually driven pharmacologic approaches to acute trauma. CNS Spectr. 10, 99–106. doi: 10.1017/S109285290001943X 15685120

[B60] Powers A. Lathan E. C. Dixon H. D. Mekawi Y. Hinrichs R. Carter S. . (2023). Primary care-based mindfulness intervention for posttraumatic stress disorder and depression symptoms among black adults: a pilot feasibility and acceptability randomized controlled trial. Psychol. Trauma 15, 858–867. doi: 10.1037/tra0001390 36265048 PMC10227868

[B61] Qi M. Chen W. Qi G. Yuan P. Hu X. Xiang J. . (2025). The predictive effect of ASD on PTSD and the factors influencing ASD and PTSD. Injury 56:112033. doi: 10.1016/j.injury.2024.112033 39602847

[B62] Roberts N. P. Kitchiner N. J. Kenardy J. Bisson J. I. (2010). Early psychological interventions to treat acute traumatic stress symptoms. Cochrane Database Syst. Rev. 2010:Cd007944. doi: 10.1002/14651858.CD007944.pub2 20238359 PMC11491193

[B63] Roberts N. P. Kitchiner N. J. Kenardy J. Lewis C. E. Bisson J. I. (2019). Early psychological intervention following recent trauma: a systematic review and meta-analysis. Eur. J. Psychotraumatol. 10:1695486. doi: 10.1080/20008198.2019.1695486 31853332 PMC6913678

[B64] Russell G. Lightman S. (2019). The human stress response. Nat. Rev. Endocrinol. 15, 525–534. doi: 10.1038/s41574-019-0228-0 31249398

[B65] Ruzek J. I. Brymer M. J. Jacobs A. K. Layne C. M. Vernberg E. M. Watson P. J. . (2007). Psychological first aid. J. Ment. Health Counsel. 29, 17–49. doi: 10.17744/mehc.29.1.5racqxjueafabgwp

[B66] Sanden S. Eid J. Hystad S. W. (2025). Predictors of acute stress disorder following a military maritime accident. Front. Psychiatry 16:1654552. doi: 10.3389/fpsyt.2025.1654552 41210123 PMC12590238

[B67] Schnurr P. P. Hamblen J. L. Wolf J. Coller R. Collie C. Fuller M. A. . (2024). The management of posttraumatic stress disorder and acute stress disorder: synopsis of the 2023 U.S. Department of Veterans Affairs and U.S. Department of Defense Clinical Practice Guideline. Ann. Intern. Med. 177, 363–374. doi: 10.7326/M23-2757 38408360

[B68] Shih C. H. Zhou A. Grider S. Xie H. Wang X. Elhai J. D. . (2023). Early self-reported post-traumatic stress symptoms after trauma exposure and associations with diagnosis of post-traumatic stress disorder at 3 months: latent profile analysis. BJPsych. Open 9:e27. doi: 10.1192/bjo.2023.1 36700253 PMC9885326

[B69] Sippel L. M. Liebman R. E. Schäfer S. K. Ennis N. Mattern A. C. Rozek D. C. . (2024). Sources of social support and trauma recovery: evidence for bidirectional associations from a recently trauma-exposed community sample. Behav. Sci. 14:284. doi: 10.3390/bs14040284 38667080 PMC11047467

[B70] Speer K. E. Semple S. Naumovski N. D'Cunha N. M. McKune A. J. (2019). HPA axis function and diurnal cortisol in post-traumatic stress disorder: a systematic review. Neurobiol. Stress 11:100180. doi: 10.1016/j.ynstr.2019.100180 31236437 PMC6582238

[B71] Stein M. B. Kerridge C. Dimsdale J. E. Hoyt D. B. (2007). Pharmacotherapy to prevent PTSD: results from a randomized controlled proof-of-concept trial in physically injured patients. J. Trauma Stress 20, 923–932. doi: 10.1002/jts.20270 18157888

[B72] Svetlitzky V. Farchi M. Ben Yehuda A. Adler A. B. (2020b). Yahalom: a rapid intervention for acute stress reactions in high-risk occupations. Mil. Behav. Health 8, 232–242. doi: 10.1080/21635781.2019.1664356

[B73] Svetlitzky V. Farchi M. Ben Yehuda A. Start A. R. Levi O. Adler A. B. . (2020a). Yahalom training in the military: assessing knowledge, confidence, and stigma. Psychol. Serv. 17, 151–159. doi: 10.1037/ser0000360 31120293

[B74] Thompson-Hollands J. Strage M. DeVoe E. R. Beidas R. S. Sloan D. M. (2021). Development and initial testing of a brief adjunctive intervention for family members of veterans in individual PTSD treatment. Cogn. Behav. Pract. 28, 193–209. doi: 10.1016/j.cbpra.2020.06.007 35967077 PMC9367094

[B75] Torres-Giménez A. Garcia-Gibert C. Gelabert E. Mallorquí A. Segu X. Roca-Lecumberri A. . (2024). Efficacy of EMDR for early intervention after a traumatic event: a systematic review and meta-analysis. J. Psychiatr. Res. 174, 73–83. doi: 10.1016/j.jpsychires.2024.04.019 38626564

[B76] Tripathi A. Brahma A. Malhotra S. Akula V. (2023). Clinical practice guidelines for assessment and management of patients presenting with psychosocial crisis. Indian J. Psychiatry 65, 212–220. doi: 10.4103/indianjpsychiatry.indianjpsychiatry_485_22 37063625 PMC10096203

[B77] Wang L. Norman I. Edleston V. Oyo C. Leamy M. (2024). The effectiveness and implementation of psychological first aid as a therapeutic intervention after trauma: an integrative review. Trauma Violence Abuse 25, 2638–2656. doi: 10.1177/15248380231221492 38281196 PMC11370167

[B78] Wang Y. Chung M. C. Wang N. Yu X. Kenardy J. (2021). Social support and posttraumatic stress disorder: a meta-analysis of longitudinal studies. Clin. Psychol. Rev. 85:101998. doi: 10.1016/j.cpr.2021.101998 33714168

[B79] Weiss D. S. (2007). “The impact of event scale: revised,” in Cross-Cultural Assessment of Psychological Trauma and PTSD, eds. J. P. Wilson and C. S.-k. Tang (Boston, MA: Springer), 219–38. doi: 10.1007/978-0-387-70990-1_10

[B80] Weiss D. S. Marmar C. R. (1997). “The Impact of Event Scale-Revised,” in Assessing Psychological Trauma and PTSD, eds. J. P. Wilson, and T. M. Keane (New York, NY: Guilford Press), 399–411.

[B81] Williams M. T. Lewthwaite H. Fraysse F. Gajewska A. Ignatavicius J. Ferrar K. . (2021). Compliance with mobile ecological momentary assessment of self-reported health-related behaviors and psychological constructs in adults: systematic review and meta-analysis. J. Med. Internet Res. 23:e17023. doi: 10.2196/17023 33656451 PMC7970161

[B82] Worku A. Tesfaw G. Getnet B. (2022). Acute stress disorder and the associated factors among traumatized patients admitted at FELEGE-HIWOT and the university of Gondar comprehensive specialized hospitals in northwest Ethiopia. BMC Psychiatry 22:309. doi: 10.1186/s12888-022-03961-9 35501782 PMC9059423

[B83] Wright S. L. Karyotaki E. Cuijpers P. Bisson J. Papola D. Witteveen A. . (2024). EMDR *v*. other psychological therapies for ptsd: a systematic review and individual participant data meta-analysis. Psychol. Med. 54, 1580–1588. doi: 10.1017/S0033291723003446 38173121

[B84] Wu M. Wang W. Zhang X. Li J. (2022). The prevalence of acute stress disorder after acute myocardial infarction and its psychosocial risk factors among young and middle-aged patients. Sci. Rep. 12:7675. doi: 10.1038/s41598-022-11855-9 35538120 PMC9091242

[B85] Yang Y. Tang J. Jiang Y. Liu X. Sun Y. Zhu X. . (2011). Development of the acute stress response scale. Soc. Behav. Pers. 39, 713–720. doi: 10.2224/sbp.2011.39.5.713

[B86] Yeo I. S. (2023). Psychiatric casualties during the Korean war: focusing on american and common wealth soldiers. Uisahak 32, 865–889. doi: 10.13081/kjmh.2023.32.865 38273723 PMC10822695

[B87] Yurtsever A. Konuk E. Akyüz T. Zat Z. Tükel F. Çetinkaya M. . (2018). An eye movement desensitization and reprocessing (EMDR) group intervention for syrian refugees with post-traumatic stress symptoms: results of a randomized controlled trial. Front. Psychol. 9:493. doi: 10.3389/fpsyg.2018.00493 29946275 PMC6005903

[B88] Zatzick D. Jurkovich G. Heagerty P. Russo J. Darnell D. Parker L. . (2021). Stepped collaborative care targeting posttraumatic stress disorder symptoms and comorbidity for US trauma care systems: a randomized clinical trial. JAMA Surg. 156, 430–442. doi: 10.1001/jamasurg.2021.0131 33688908 PMC7948109

[B89] Zhengzhi F. Jia W. (2019). Research progress and prospect of combat stress response. J. Third Mil. Med. Univ. 41, 275–281. doi: 10.16016/j.1000-5404.201809150

